# The Influence of Teachers on Motivation and Academic Stress and Their Effect on the Learning Strategies of University Students

**DOI:** 10.3390/ijerph17239089

**Published:** 2020-12-05

**Authors:** Rubén Trigueros, Ana Padilla, José M. Aguilar-Parra, María J. Lirola, Amelia V. García-Luengo, Patricia Rocamora-Pérez, Remedios López-Liria

**Affiliations:** 1 Department of Psychology, Hum-878 Research Team, Health Research Centre, University of Almería, 04120 Almería, Spain; rtr088@ual.es; 2 Research Center Háblame, 04005 Almería, Spain; anapadilla@centrohablame.com; 3 Department of Education, University of Almería, 04120 Almería, Spain; mariajesus.lirola@ual.es; 4 Department of Mathematics, University of Almería, 04120 Almería, Spain; amgarcia@ual.es; 5 Department of Nursing, Physiotherapy and Medicine, Health Research Centre, University of Almería, 04120 Almería, Spain; rll040@ual.es

**Keywords:** academic motivation, stress, teacher, metacognitive strategies, critical thinking

## Abstract

Students often experience the university period as a very stressful time. The teacher is a key figure who can cushion this stressful experience for the student. This study therefore aims to analyse the influence of teachers from the Self-Determination Theory perspective on academic stress, motivation, critical thinking, metacognitive strategies and academic performance in university students. The study involved 2456 university students with an average age of 22.51 years. A structural equation model was created to analyse the causal relationships between the variables. The results showed that the psychological controlling of the teacher positively predicted academic stress while autonomy support negatively predicted academic stress. Academic stress negatively predicted motivation, metacognitive strategies, critical thinking and academic performance. Academic motivation positively predicted metacognitive strategies and critical thinking. Finally, metacognitive strategies and critical thinking positively predicted academic performance. These results highlight the importance of the role that the teacher adopts during classes and the protective factor of academic motivation in the presence of stress.

## 1. Introduction

The university educational context often creates stress and anxiety among students due to the competitive environment it usually generates [1]. This causes an overwhelming emotional burden that leaves students with minimal opportunity to relax and recreate, which can negatively affect them in performing tasks effectively and consequently affect their academic performance [2,3]. In this sense, the role that teachers play can be extremely important towards the development of positive attitudes that generate a climate oriented towards living a series of experiences that promote personal abilities, fun, self-knowledge, internal motivation, transference and learning [4]. Thus, this study aims to analyse the influence of teachers from the Self-Determination Theory perspective on academic stress, motivation, critical thinking, metacognitive strategies and academic performance in university students.

### 1.1. Self-Determination Theory

The Self-Determination Theory (SDT) is a macro theory of human motivation that suggests the significant influence of the social context on human behaviour [5]. In this sense, SDT states that the teacher’s influence can be supportive of autonomy versus psychological control [6,7]. Teacher support for student autonomy is a basic teaching feature that encourages self-determined motivation and student engagement in the classroom [7]. In this sense, by promoting personal initiative, self-regulation, self-concept, and by offering relevant objectives and the use of minimum contingencies, the teacher will encourage support for student autonomy. On the contrary, the teacher’s psychological control over students encourages controlled motivation and hinders the student’s commitment in the classroom [7]. To this end, the use of contingencies to guide behaviour and excessively light or demanding objectives will involve the psychological control of the student.

Furthermore, SDT affirms the existence of a motivational continuum that goes from autonomous motivation to demotivation, as well as controlled motivation [8,9]. In this sense, self-determined motivation includes behaviours related to self-choice, the ability to make one’s own decisions and personal initiative. On the other hand, controlled motivation is related to external pressures and acquired obligations (either to oneself or to another person). This type of motivation brings with it a lack of self-regulation of adaptive behaviour and a tendency to desist and withdraw from action due to the absence of rewards or social recognition [9]. However, autonomous motivation facilitates the adaptation of the individual to the context because they tend to manifest behaviours related to persistence and adhere to behaviours related to enjoyment, which come from personal improvement and the sensations inherent to the activity itself [10].

Most studies that have existed in the field of SDT up to now have only analysed how teacher autonomy support and psychological control were related to student motivation [11,12,13] and basic psychological needs [14], and there have been few studies that have analysed the relationship between teacher behaviour and student psychological distress, especially those related to stress. In this sense, the teacher can be a good channeler and attenuator of the negative feelings and emotions experienced by students, due to the influence they have on them as a result of their knowledge of the subject and daily interaction with students.

### 1.2. Academic Stress

Stress has been shown to be very present throughout the academic period of university students, especially in the first years of the degree course and during specific periods such as exams, paper presentations or oral presentations [15]. From the university academic context, this stress is known as academic stress and is defined as a psychological state of the individual generated by continuous social and personal pressure that produces a depletion of the individual’s reserves [16]. Such a state produces in the student an inability to adapt to the demands that are present in the university academic environment [17,18]. Academic stress is not only found in the university environment however, but is specific to each of the academic stages (e.g., secondary, primary and infant), although it is during the university stage that the stress reaches its highest point due to the high demands of this stage [2,19].

Studies on academic stress in the university setting are generally scarce [20]. Different studies have shown the existence of notable indices of stress in university populations, reaching higher levels in the first years of study and in the periods immediately prior to exams [21], and being lower in the last years of study. Similarly, studies have shown that academic stress has been positively associated with poor academic performance [22], school failure [23] and depression [24]. However, few studies have analysed how academic stress has been related to students’ academic motivation and their use of learning strategies.

### 1.3. Metacognitive Strategies and Critical Thinking

Traditionally, and today, the teaching methods that predominate in schools are based on a limited practical applicability of knowledge and a pre-eminence of rote learning [25]. Thus, students are faced with fictional situations designed by the teacher that often differ from real situations [26]. Furthermore, the education system often pays more attention to the results achieved by students, without worrying about the mental processes they have used to assimilate information and/or whether there is a clear applicability of the assimilated knowledge by students to various everyday situations [27]. For this reason, one of the aspects that must be promoted from the educational point of view is the predominance of significant learning, with the aim of students developing conscious and reflexive learning that will allow them not only to consolidate their own knowledge but also to establish the information, skills and values that they will have to assimilate and apply in the future [28].

Thus, metacognitive strategies applied in the educational field can help to develop greater mental activity in the student, as they allow them to observe their own learning process, using various resources that serve to plan, control and evaluate their own progress [27]. In this way, the student develops a much more complex mental activity that can lead them to reassess their way of thinking, knowledge and beliefs about themselves and the context around them [29].

Studies focusing on metacognitive strategies in education have shown a positive relationship with respect to academic performance [19,30], achievement of learning objectives [31], reading comprehension [32] and second-language learning [33].

### 1.4. Objectives and Hypothesis

The aim of this study is to analyse the influence of teachers from the SDT perspective on academic stress, motivation, critical thinking, metacognitive strategies and academic performance in university students. The following hypotheses are specified: (a) Psychological controlling by the teacher will positively predict academic stress. However, autonomy support will predict it negatively. (b) Academic stress will negatively predict motivation, metacognitive strategies, critical thinking and academic performance. (c) Academic motivation will positively predict metacognitive strategies, critical thinking and academic performance. (d) Metacognitive strategies and critical thinking will positively predict academic performance.

## 2. Methodology

### 2.1. Participants

This study involved 2456 university students (1358 women and 1098 men), belonging to the University of Almeria and Malaga. The age of the participants varied between 19 and 26 years (M = 22.45; SD = 1.45).

### 2.2. Measurement

#### 2.2.1. Perceived Autonomy Support

The short version of the Teacher as Social Context Questionnaire [34] was used. The scale is composed of a single factor with 8 items. The students responded through a Likert-type scale that ranged from completely disagree (1) to completely agree (7). The reliability analysis of the original scale revealed a score of 0.79. 

#### 2.2.2. Psychological Control

The Spanish version of the scale [35] from the psychologically controlling teaching scale [36] was used. The scale is composed of a single factor with 7 items. The students responded through a Likert-type scale that ranged from totally disagree (1) to totally agree (5). The reliability analysis of the original scale revealed a score of 0.82.

#### 2.2.3. Academic Stress

The inventory stress manifestations [37] for students were used, specifically the Spanish version [38]. The scale is composed of 22 items that are divided into three factors: physiological (6 items), emotional (10 items) and behavioural 6 items). The students responded through a Likert-type scale that ranged from not at all (1) to totally agree (5). The reliability analysis of the original scale revealed a score of 0.81.

#### 2.2.4. Academic Motivation towards Learning

The scale was used from the Spanish version [39] of the Academic Self-Regulation Scale by Vansteenkiste, Sierens, Soenens, Luyckx and Lens [40]. This scale is made up of six factors, namely intrinsic motivation (four items), integrated regulation (four items), identified regulation (four items), introjected regulation (four items), external regulation (four items) and de-motivation (four items). The students responded to each of the items using a Likert-type scale ranging from 1 (not true) to 7 (completely true).

To assess the level of academic motivation, the self-determination index was calculated [41]. This index has been shown to be reliable and valid in numerous studies [42,43].

#### 2.2.5. Metacognitive Strategies and Critical Thinking

The questionnaire for Spaniards [44] from the Motivated Strategies for Learning Questionnaire [45] was used. Specifically, only the 12 items that measure metacognitive strategies and the 5 items for critical thinking were taken into consideration. Students responded to each of the items using a Likert-type scale ranging from 1 (not true) to 5 (completely true). The reliability analysis of the original scale revealed scores of 0.82 (metacognition strategies) and 0.80 (critical thinking).

#### 2.2.6. Academic Performance

The average grades of the students at the end of the 2019/2020 academic year were considered to analyse their academic performance. The grades ranged from 5 (outstanding) to 1 (failing).

### 2.3. Procedure

Before starting the study, the heads of faculty and teaching staff were contacted, asked for their collaboration and informed of the objectives of the study. Once their approval had been obtained, students were required to participate in the study by signing an informed consent form, which contained information about the study in which they were to participate. The questionnaires were completed by the students individually, anonymously and at the beginning of the classes. In addition, a member of the study was present to answer any questions that the study participants might have regarding the questionnaires.

Finally, it should be noted that this study has followed the guidelines recommended by the American Psychological Association and obtained its ethical approval by the Research Bioethics Committee of the University of Almeria (Ref. UALBIO 2019/014).

### 2.4. Data Analysis

In the present study, several descriptive statistical analyses (e.g., mean, standard deviation and Pearson’s bivariate correlations) and reliability analyses have been carried out. The SPSS v.25 statistical package (IBM, Armonk, NY, USA) was used for these analyses. In addition, a structural equation model (SEM) was used to analyse the predictive relationships established in the hypothesised model. The AMOS v.20 statistical package (IBM, Armonk, NY, USA) was used for this analysis. For SEM, the maximum likelihood procedure was followed together with a bootstrapping of 6000 interactions. The adjustment rates for accepting a rejection of the model are listed in Table 1 [46].

These rates must be interpreted with caution, as they are too strict or complicated to achieve in complex models [47].

## 3. Results

### 3.1. Preliminary Analysis

The bivariate correlations, as shown in Table 2, were positive between each of the study variables, except those related to psychological control and academic stress. Finally, it should be noted that the reliability analysis showed a score higher than 0.70 [48] so that each of the factors was considered reliable.

### 3.2. Structural Equation Modelling

**Figure 1:**
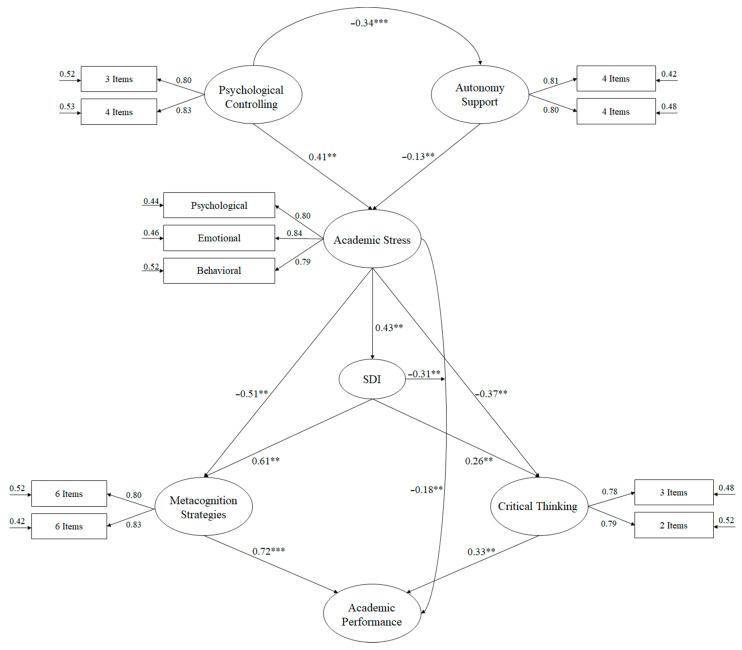
Structural equation model where the causal relationships between the variables of the study are established. Note: *** *p* < 0.001; ** *p* < 0.01. Self-Determination Index

The adjustment rates of the hypothesised model, through the analysis of structural equations (Figure 1), to analyse the predictive relationships were adequate: χ^2^ (55, *n* = 2456) = 152.65, χ^2^/df = 2.77, *p* < 0.001, IFI = 0.97, TLI = 0.97, CFI = 0.97, RMSEA = 0.059 (IC 90% = 0.051–0.063), SRMR = 0.042. These indexes reflect that the model had an acceptable adjustment and should therefore be considered as adequate. Standardised regression was used to analyse the relationships between the study variables.

The relationships established in the structural equation model are specified below:

(a) Teacher control has been positively related to academic stress (β = 0.43, *p* < 0.01), while autonomy support has been negatively related to academic stress (β = −0.13, *p* < 0.001).
(b) Academic stress has been negatively related to motivation (β = −0.43, *p* < 0.01), metacognitive strategies (β = −0.51, *p* < 0.01), critical thinking (β = −0.37, *p* < 0.01) and academic performance (β = −0.18, *p* < 0.01).
(c) Academic motivation has been positively related to metacognitive strategies (β = 0.61, *p* < 0.01) and critical thinking (β = 0.26, *p* < 0.01).
(d) Metacognitive strategies and critical thinking will positively predict academic performance (β = 0.72, *p* < 0.001) and (β = 0.33, *p* < 0.01) respectively.

## 4. Discussion

The present study aimed to analyse the relationship from the duality of teacher influence (autonomy support vs. psychological control) from the perspective of SDT, on academic stress, motivation, critical thinking, metacognitive strategies and academic performance in university students. This study considers for the first time the direct relationship between teachers’ behaviours and students’ psychological distress in the academic environment. In this sense, teachers, as managers of the academic programme of their subjects, can have a decisive influence on the perception and emotions experienced by students [49]. In addition, this study looks for the first time at how students’ psychological distress influences their own motivation, metacognitive learning strategies and critical thinking. In this sense, psychological distress has been positively related to poor academic performance, with a poor adaptive process on the part of students to academic demands [50,51]. 

The results of the present study showed that autonomy support predicted negatively with academic stress, while psychological control predicted positively with academic stress. Despite the results achieved, no studies have been found that address these variables from the university academic environment. However, SDT suggests that autonomy support promotes the process of adaptation of students to the possible vicissitudes they may face by making their own decisions [52,53]. On the contrary, the psychological control exercised by the teacher interferes with the students’ decision-making process, negatively affecting their adaptation to these vicissitudes, since the possible decisions taken by the students are the result of the decisions made by the teacher [7,52]. Depending on the type of influence exerted by the teacher, this may generate a greater or lesser level of stress, since students could use coping strategies that they already know, favouring their adaptation process in the face of vicissitudes. The university academic context is an enormous psychological and physical strain for the student, due to the constant dedication required and constant challenges faced [54]. Therefore, depending on the influence that the teacher exerts, they may increase or decrease the feeling of stress in the face of academic challenges and demands that students face, influencing students’ coping strategies [6,14]. In this way, students with an advantage in autonomy will develop their own coping strategies that they are more comfortable with [52]. On the other hand, if they feel controlled by the teacher, they will not develop coping strategies to deal with stressful situations, as the teacher is the one who makes decisions without time to reflect and learn from their mistakes [6,7].

The results of the present study showed that academic stress predicted negatively with academic motivation, learning strategies, critical thinking and academic performance. Despite the results achieved, no studies have been found that address these variables from the academic sphere, only in an isolated manner [55,56,57]. In this regard, a study conducted by Park et al. [56] with university medical students found that academic stress was a negative predictor of motivation and academic performance. Similarly, a study by Karaman and Watson [58] of high school students showed that those students who had high levels of stress had low levels of internal motivation and low life satisfaction. Despite the results of the present study, there are hardly any studies in the academic field that have linked academic stress to the metacognitive strategies and critical thinking of university students. A study by Roussis and Wells [59] showed that students with high levels of stress made little use of metacognitive strategies and used rote learning strategies instead in order to regain control over their academic performance. On the other hand, a study conducted by Yuan, Zhang and Fu [60] with high school students showed that those students who were facing very stressful academic situations showed a superficial style of thinking, while those students whose stress level was medium–high showed critical thinking, which favoured their adaptation to vicissitudes. In this way, the presence of academic stressors can be detrimental to an individual’s adaptation. This implies a loss of interest in the acquisition of new skills, abilities and knowledge related to the use of those strategies aimed at the realisation of comprehensive and meaningful learning, which are related to obtaining good academic results. Therefore, it is necessary to limit such experiences and promote a more comprehensive education without emphasising only academic performance.

On the other hand, results have shown that motivation, critical thinking and academic stress positively predicted academic performance. Furthermore, motivation moderated the effects of academic stress on academic performance, mitigating the negative effects of stress. These results have been described in research with university populations [61], high school students [62] and distance learning students [63]. These findings could be explained by the fact that metacognitive strategies are procedures that facilitate information processing by selecting, organising and regulating cognitive processes [64]. In order to use them, students need to show a strong interest in the subject (i.e., an internal motivation towards it), as it requires a conscious planning and use of these strategies, which facilitate academic performance. In the same way, critical thinking requires a high degree of effort on the part of students, who are only willing to invest if academic achievement is attainable [62]. Therefore, students will be willing to make an effort to think critically if they perceive a high degree of control over their academic achievement. While the relationship between a readiness to think critically and metacognition strategies with respect to academic achievement seems quite logical, there are few studies that have taken this relationship into account. In this sense, Hall, Hladkyj, Perry and Ruthig [65] found that academic success in university students was positively related to the use of elaborate learning (e.g., paraphrasing, summarising), which is a learning strategy that is highly correlated with the disposition of critical thinking.

In spite of the results achieved in this study, it is important to point out a series of limitations: (a) the study has been based on the use of self-reported questionnaires, which limits the access to information of the subjects; (b) the interpretation of the relationships between the variables of the present study can be interpreted differently according to the opinion of each reader; (c) the selection of the participants in the study has been non-probabilistic and, therefore, comparisons between the populations of the study cannot be established. For all these reasons, future studies that follow a mixed methodology (quantitative and qualitative) should deepen the relationships between academic stress and learning strategies of university (and non-university) students, as well as incorporating other factors such as resilience, emotions and coping strategies.

## 5. Conclusions

The results of the present study have shown how the psychological control exercised by the teacher positively predicted academic stress, while the autonomy support negatively predicted academic stress. Academic stress negatively predicted motivation, metacognitive strategies, critical thinking and academic performance. Academic motivation positively predicted metacognitive strategies, critical thinking and academic performance. Finally, metacognitive strategies and critical thinking positively predicted academic performance. Due to these results, it is necessary to implement educational programmes that enrich the class dynamics that take place at university, so that students feel more motivated and can channel academic stress in a more positive way.

## Figures and Tables

**Table 1:** Adjustment indexes.

Statistics	Good Indexes
Chi square/degree freedom	Between 2 and 3
Comparative Fit Index (CFI)	Greater than 0.95
Incremental Fit Index (IFI)	Greater than 0.95
Tucker Lewis Index (TLI)	Greater than 0.95
Root-Mean-Square Error of Approximation (RMSEA) and its 90% confidence interval	Equal or less than 0.06
Standardized Root-Mean-Square Residual (SRMR)	Equal or less than 0.08

**Table 2:** Descriptive statistics and correlations between all variables.

Factors	*M*	*SD*	α	1	2	3	4	5	6	7
1. Autonomy Support	5.18	1.99	0.81	-	−0.38 ***	−0.49 ***	0.39 ***	0.47 ***	0.12 *	0.43 ***
2. Psychological Control	1.97	1.65	0.83		-	0.28 **	−0.51 ***	−0.51 ***	−0.32 **	−0.46 **
3. Academic stress	2.42	1.88	0.87			-	−0.61 **	−0.33 **	−0.08	−0.55 ***
4. SDI-Academic	15.37	8.46	-				-	0.42 ***	0.47 **	0.38 **
5. Metacognition Strategies	4.13	0.74	0.80					-	0.52 ***	0.53 ***
6. Critical Thinking	3.88	0.87	0.79						-	0.46 ***
7. Academic Performance	2.84	1.03	-							-

Note: SDI = Self-Determination Index; *** *p* < 0.001; ** *p* < 0.01; * *p* < 0.05
